# Asymmetric masks for laboratory-based X-ray phase-contrast imaging with edge illumination

**DOI:** 10.1038/srep25466

**Published:** 2016-05-05

**Authors:** Marco Endrizzi, Alberto Astolfo, Fabio A. Vittoria, Thomas P. Millard, Alessandro Olivo

**Affiliations:** 1 Department of Medical Physics and Biomedical Engineering, University College London, Gower Street, London WC1E 6BT, United Kingdom

## Abstract

We report on an asymmetric mask concept that enables X-ray phase-contrast imaging without requiring any movement in the system during data acquisition. The method is compatible with laboratory equipment, namely a commercial detector and a rotating anode tube. The only motion required is that of the object under investigation which is scanned through the imaging system. Two proof-of-principle optical elements were designed, fabricated and experimentally tested. Quantitative measurements on samples of known shape and composition were compared to theory with good agreement. The method is capable of measuring the attenuation, refraction and (ultra-small-angle) X-ray scattering, does not have coherence requirements and naturally adapts to all those situations in which the X-ray image is obtained by scanning a sample through the imaging system.

X-ray phase contrast imaging (XPCI)^1^,^2^ extends the potential of conventional, attenuation based X-ray imaging by providing additional contrast mechanisms arising from the phase-shifts imparted to the beam by the sample. Its potential applications span across a variety of fields, as diverse as medicine, materials science, security screening and biology. Amongst various solutions proposed^3^,^4^,^5^,^6^,^7^,^8^,^9^,^10^,^11^,^12^,^13^, we focus here on edge illumination (EI)^14^ for its capability to provide quantitative attenuation, phase^15^ and scattering^16^ information with a setup that uses commercial rotating anode source and detector technology. EI was initially developed by using monochromatic synchrotron radiation, then implemented with conventional rotating anode and microfocal X-ray tubes^17^,^18^, thanks to its negligible requirements in terms of spatial or temporal coherence^19^,^20^, its high sensitivity^21^,^22^ and its robustness against thermal and mechanical instabilities^23^,^24^.

Re-positioning of the optics and/or of the sample during data acquisition is typically required in XPCI experiments in order to separate phase and attenuation contributions to the measured intensity projections. Limiting ourselves to those methods capable of tolerating extended X-ray tube sources, potential solutions involve a grating interferometer scanning set-up^25^,^26^, magnetic phase stepping^27^ or Fourier fringe analysis methods^28^,^29^ which however provide a reduced spatial resolution. Another approach, where an asymmetric design could potentially be incorporated, is a non-scanning grating interferometer set-up^30^.

We introduce an approach based on the use of an asymmetric mask that eliminates all movements typically associated with EI XPCI experiments, with exception of the sample which is scanned through the imaging system. Sample scanning also removes sampling problems that might be encountered when imaging an object through a small aperture^31^. The basic idea is that an asymmetric pattern of apertures and absorbing septa are designed in such a way that adjacent detector pixel columns receive different degrees of illumination. The intensity projections measured by adjacent columns are then combined to retrieve the sample attenuation, refraction and scattering. This results in an XPCI system that does not require any re-arrangement of the optics during data acquisition. Moreover, if masks large enough to cover the entire object are available and a loss in resolution can be tolerated, the need to scan the object is also eliminated, and data acquisition becomes completely stationary.

## Methods

**Figure 1:**
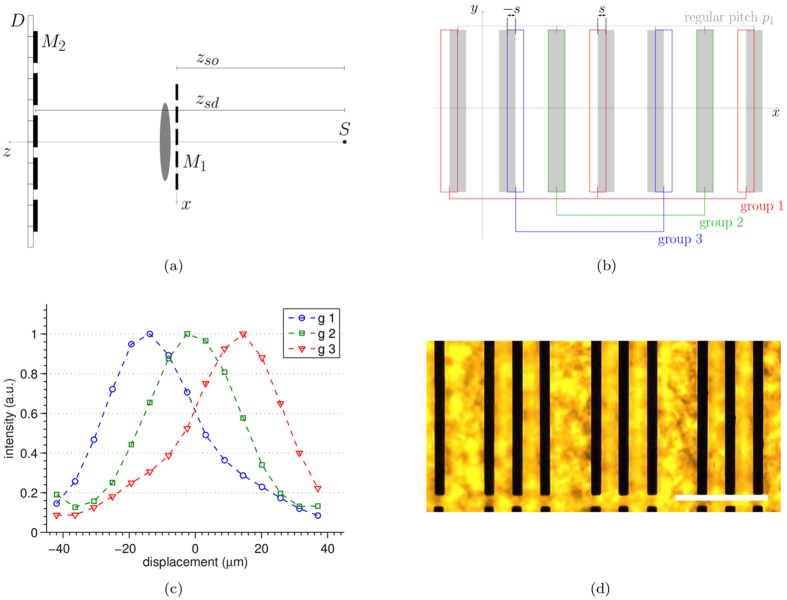
(**a**) sketch of the experimental set-up: the X-rays generated by an extended source *S* are shaped by the sample mask *M*_1_, propagate through the sample and are analysed by the detector mask *M*_2_ before reaching the detector *D*. (**b**) 3-way asymmetric mask design: grey rectangles represent the standard aperture pattern (detector mask) while the coloured rectangles indicate the asymmetric aperture pattern (sample mask). (**c**) Experimental illumination functions corresponding to each aperture group. Each group is shifted with respect to the others by the known amount *s*. The three complementary EI conditions (i.e. ±60% and 100% illumination) can be simultaneously acquired at the mask position corresponding to 0 *μ*m displacement. (**d**) optical microscope image of the 3-way asymmetric mask manufactured for this experiment, scale bar is 200 *μ*m.

The set-up for laboratory-based EI experiments consists of a rotating anode source generating an X-ray beam that is shaped by a mask, passes through the object under investigation, and is then analysed by a second mask placed in front of the detector (see Fig. 1(a)). The first mask *M*_1_ is generally referred to as the sample mask and the second *M*_2_ as the detector mask. The masks are designed in such a way that the magnified sample mask pitch *Gp*_1_ is equal to the detector mask pitch *p*_2_, where *G* = *z*_*sd*_/*z*_*so*_ is the geometrical magnification given by the ratio between the source-to-detector *z*_*sd*_ and the source-to-object *z*_*so*_ distances. The detector mask pitch *p*_2_, in turn, matches the detector pixels’ pitch *p*_3_ and a one-to-one relationship exists between each aperture and detector pixel column. The typical design is that of a periodic, harmonically matched set of apertures and absorbing septa which provides a uniform illumination level across the entire field of view. The variation in the detected intensity as a function of the lateral shift between sample and detector mask along *x*, the direction orthogonal to both the beam propagation and the apertures, is described by the illumination function (see Fig. 1(c)). In a standard and correctly aligned EI system^23^ all pixels detect the same illumination function, with no relative shifts between the curves recorded by different pixels.

A 3-way asymmetric mask is obtained through a modification of the conventional mask design used in EI^17^. As shown in Fig. 1(b), group 1 and group 3 apertures are shifted by ±*s* with respect to the position of group 2 apertures, which is the same as in the conventional design. This can also be seen as three regularly spaced groups of apertures, each with period 3*p*_1_, shifted by ±*s* with respect to each other. When a sample mask with such design is scanned along *x*, each detector column records one of the intensity curves shown in Fig. 1(c). These are in fact three illumination functions, shifted with respect to each other by ±*s*. Three independent intensity projections are obtained by each group of pixel columns, for example with the mask in the position indicated by 0 *μ*m displacement in Fig. 1(c). These are then combined to retrieve the absorption, refraction and scattering properties of the object. The change in shape between the illumination functions of group 1, 2 and 3 that can be observed in Fig. 1(c) is the result of the cross-talk between adjacent pixels. A comprehensive discussion of this effect can be found in the Supplementary Information online. It should be noted, however, that these changes in shape do not represent a practical problem because this information can be explicitly used in the retrieval, leading to quantitatively accurate results^24^.

This 3-way asymmetric mask concept can be extended to any desired number *m*, where the apertures of a standard mask design are classed in *m* groups, each one positioned with a different relative shift. In order to demonstrate this, a 7-way asymmetric mask was designed and experimentally tested. In this case the asymmetric masks were designed to produce the same illumination level, with opposite slopes, in pairs of apertures belonging to different groups. In this way existing retrieval algorithms^16^,^32^ could be used. However, it should be noted that this restriction is not necessary, and that the relative shifts between groups of apertures can be optimised depending on the specific application.

For a sample of known shape and composition, attenuation profiles are modelled in the following way^33^:


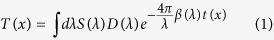


where *λ* is the radiation wavelength, *S*(*λ*) is the spectral distribution of the X-ray source, *D*(*λ*) is the energy response function of the detector, 4*πβ*(*λ*)/*λ* is the linear attenuation coefficient of the material with *β* the imaginary part of the refractive index *n* = 1 − *δ* + *iβ*, and *t*(*x*) is the projected thickness of the sample along the beam propagation axis. The detected transmission is then modelled through a convolution:


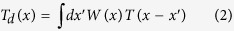


where *W*(*x*) = *rect*(*x*/*a*_1_), *rect*(*x*/*a*_1_) is the rectangular function defined as 1 for −1/2 < *x* < 1/2 and 0 elsewhere, and *a*_1_ is the width of the apertures in the sample mask. This accounts for the fact that we are illuminating the sample by scanning it through an X-ray beam shaped by a rectangular window. The refraction profiles are modelled as:


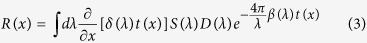


and, as for the attenuation case, the detected signal is modelled through a convolution *R*_*d*_(*x*) = (*W* ∗ *R*)(*x*).

Three objects were imaged. The first was a custom built phantom made of fibres of various materials with nominal properties as follows: Nylon of 100 and of 300 *μ*m diameter, Sapphire of 250 *μ*m diameter and PEEK of 150 *μ*m (all from Goodfellow Cambridge Ltd., England, UK). This was used for the quantitative analysis. The second phantom consisted of an acrylic rod and a paper step wedge for qualitative retrieval of mixed absorption, refraction and scatter images. Finally, a ground beetle was also imaged as an example of a more complex biological sample.

A Mo target rotating anode source (MM007, Rigaku, Japan) operated at 45 kV/20 mA was used for this experiment. The sample mask was placed at 160 cm from the source, and the detector mask an additional 40 cm downstream. The apertures were *a*_1_ = 23 *μ*m and *a*_2_ = 29 *μ*m wide in the sample and detector mask, respectively. The detector mask pitch *p*_2_ was 98 *μ*m and the sample mask pitch (of each aperture group) 3*p*_1_ was 237 *μ*m. For the 3-way asymmetric sample mask, the relative shift ±*s* was ±15 *μ*m, while a set of shifts equal to (±8, ±12, ±17) *μ*m was used for the 7-way asymmetric mask. The absorbing septa were made of gold with a nominal thickness of 200 *μ*m and supported by a graphite substrate. The masks were manufactured to the authors’ design by Creatv MicroTech Inc. (Potomac, MD, US). For the two custom built phantoms, the detector was a CMOS flat panel coupled with directly deposited CsI scintillator and a pixel size of 50 *μ*m × 50 *μ*m (C9732DK-11, Hamamatsu, Japan). A photon counter employing CdTe-CMOS sensor technology and with a pixel size of 100 *μ*m × 100 *μ*m (XC-FLITE FX1, XCounter, Sweden) was used for the acquisition of the ground beetle. The CMOS flat panel sensor was used with a line-skipping mask design^34^ in order to minimise the effect of cross-talk between the pixels due to the presence of long tails in the detector point spread function. The single-photon-counting detector was used with the conventional mask design, where each pixel column is matched with an aperture in the masks. For the calculations of the theoretical profiles we used the source energy spectrum *S*(*λ*) provided by the manufacturer, a model detector response *D*(*λ*) for indirect X-ray detectors based on the amount of energy deposited into the scintillator^35^ and the values for *β*(*λ*) and *δ*(*λ*) provided by xraylib^36^.

## Results

**Figure 2:**
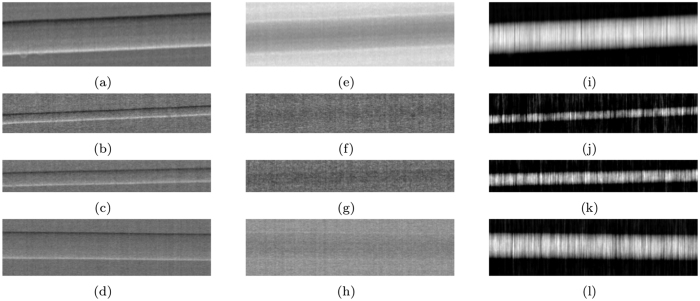
Images of the multi-wire phantom: (**a–d**) refraction, (**e–h**) attenuation and (**i–l**) phase. From top to bottom: sapphire 250 *μ*m, Nylon 100 *μ*m, PEEK 150 *μ*m and Nylon 300 *μ*m diameter. The vertical stripes that can be seen in panels (**i–l**) run parallel to the direction of integration and are a result of the presence of noise in the integrand images (**a–d**).

**Figure 3:**
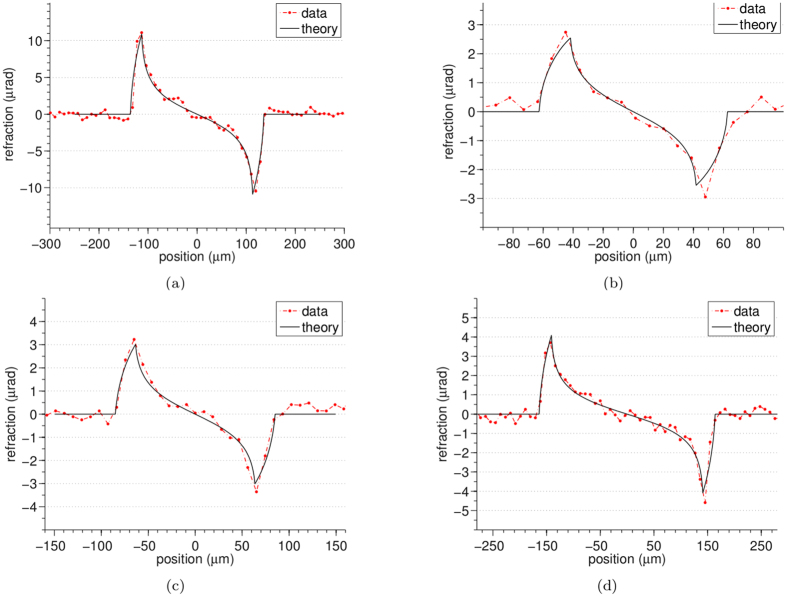
Profiles extracted from the refraction images of the multi-wire phantom: (**a**) sapphire 250 *μ*m, (**b**) Nylon 100 *μ*m, (**c**) PEEK 150 *μ*m and (**d**) Nylon 300 *μ*m diameter.

**Figure 4:**
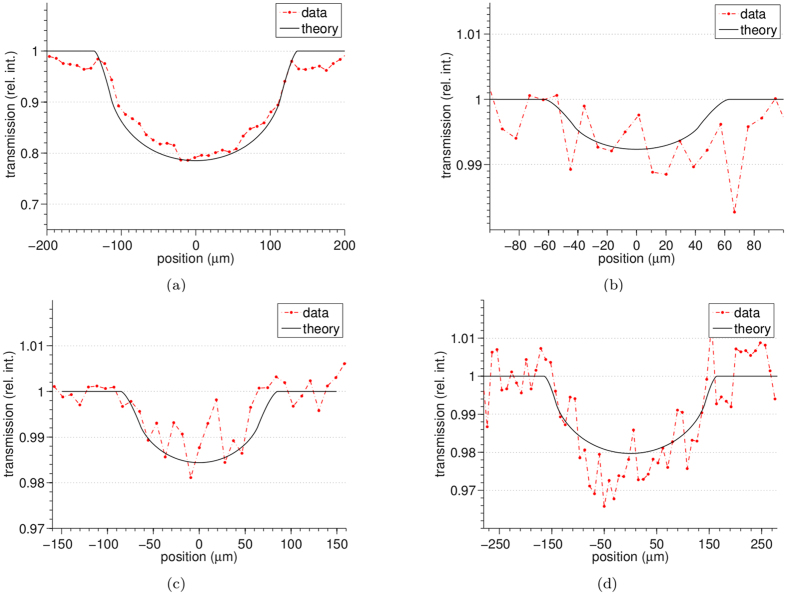
Profiles extracted from the attenuation images of the multi-wire phantom: (**a**) sapphire 250 *μ*m, (**b**) Nylon 100 *μ*m, (**c**) PEEK 150 *μ*m and (**d**) Nylon 300 *μ*m diameter.

The quantitative accuracy of the method was tested on the multi-wire sample using the 3-way mask design, with results shown in Fig. 2, for the refraction, attenuation and phase images. The phase images of Fig. 2(i–l) are obtained by numerical integration and imposing a zero phase shift in background. The vertical stripes that can be seen in the phase images of Fig. 2(i–l) are artefacts that can be attributed to the numerical integration. The refraction images in Fig. 2(a–d) are affected by random fluctuations generated by the propagation of the statistical counting noise inherent in the recorded intensity projections. After numerical integration these fluctuations results into stripe artefacts that run parallel to the direction of integration. Profiles extracted from the panels in Fig. 2(a–d,e–h) are compared to theory in Figs 3 and 4. A good agreement can be observed between the experimental (dashed red line) and the theoretical (solid black line) profiles. A relatively high level of noise can be observed in the profiles extracted from the attenuation images (Fig. 4), this is due to the weak absorption and therefore weak signal (a few percent) of these samples leading to a small signal to noise ratio.

**Figure 5:**
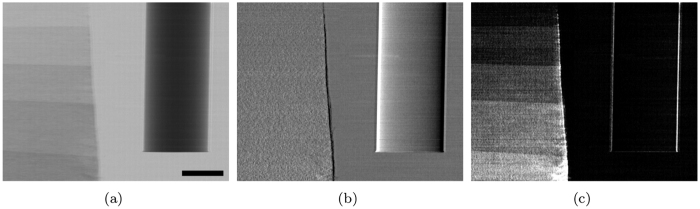
(**a**) attenuation, (**b**) refraction and (**c**) scattering contrast images of the acrylic rod and paper step wedge sample. Scale bar is 2 mm.

**Figure 6:**
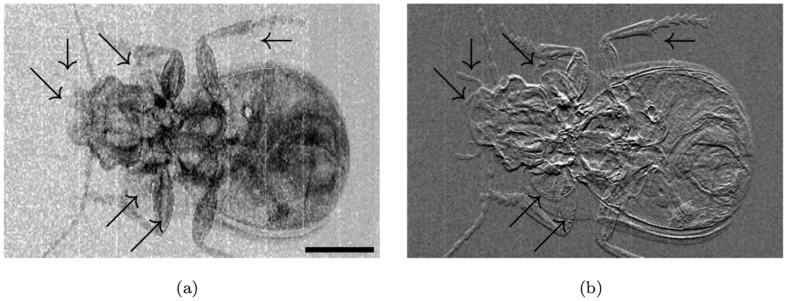
(**a**) attenuation and (**b**) refraction contrast images of the ground beetle. Scale bar is 3 mm.

Figure 5 shows the simultaneously retrieved attenuation (panel 5(a)), refraction (panel 5(b)) and scattering (panel 5(c)) images of the acrylic rod and paper step wedge sample. The dependence of the signal on the thickness of the scatterer can be observed in panel 5(c). A bright signal at the edges of the acrylic rod can also be observed, which is a known feature of this type of contrast channel^37^,^38^,^39^. The attenuation and refraction images of the ground beetle are shown in Fig. 6; the scattering image is not shown in this case due to the lack of dark-field signal for this sample. Several details that are clearly visible in the refraction image but not in the attenuation image, are indicated with arrows. This highlights the superiority of phase-contrast imaging for the visualization of faint details in biological samples.

**Figure 7:**
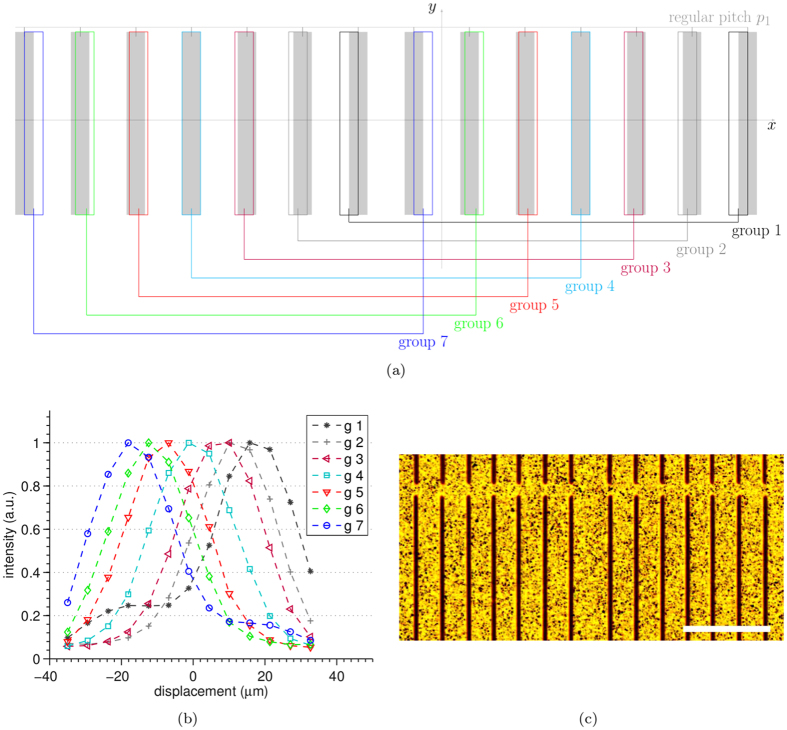
7-way asymmetric aperture pattern. (**a**) schematic of the design, (**b**) experimental illumination functions and (**c**) optical microscope image, scale bar is 250 *μ*m. At the mask position corresponding to 0 *μ*m displacement, seven complementary illumination conditions ±35%, ±60%, ±80% and 100% are obtained.

Finally, the set of illumination functions measured using the 7-way asymmetric aperture pattern is shown in Fig. 7. By placing the sample mask at position 0 *μ*m along the *x* axis, intensity projections are acquired at approximately ±35%, ±60%, ±80% and 100%. This demonstrates the possibility to implement the new mask concept proposed here also in those cases where a finer sampling of the illumination function is required by a particular application. For example, should the alignment of the entire field of view be severely limited, a larger number of intensity projections could be beneficial in order to minimise the errors in the retrieval^24^.

## Discussion

A new concept of asymmetric masks was introduced to enable laboratory-compatible X-ray phase contrast imaging with a stationary system. Attenuation, refraction and scattering were retrieved without any movement of the instrumentation during data acquisition, aside from sample scanning. Two proof-of-principle masks were designed, fabricated and experimentally tested. One was a 3-way mask, for the simultaneous acquisition of three complimentary illumination conditions, which is the theoretical minimum for the retrieval of a three-channel (attenuation, refraction and ultra-small-angle scattering) representation of the sample. The other was a 7-way mask that can be used when a finer sampling of the illumination function is required by certain classes of applications. The method was quantitatively tested on a custom built phantom made of known materials, with good agreement between experimental data and theoretically expected profiles. The potential in terms of image quality was demonstrated also on more complex samples: one which produces a non-negligible scattering signal and a biological one. This new approach preserves the incoherence and achromatic properties of edge illumination and removes any possible problem related to aliasing which might arise from incomplete sample illumination. It naturally adapts to those situations in clinical, industrial and security imaging where the image is acquired by scanning the sample through a stationary imaging system.

## Additional Information

**How to cite this article**: Endrizzi, M. *et al.* Asymmetric masks for laboratory-based X-ray phase-contrast imaging with edge illumination. *Sci. Rep.* **6**, 25466; doi: 10.1038/srep25466 (2016).

## Supplementary Material

Supplementary Information

## References

[b1] Bravin A., Coan P. & Suortti P. X-ray phase-contrast imaging: from pre-clinical applications towards clinics. Phys. Med. Biol. 58, R1 (2013). 23220766 10.1088/0031-9155/58/1/R1

[b2] Wilkins S. *et al.* On the evolution and relative merits of hard x-ray phase-contrast imaging methods. Phil. Trans. R. Soc. A 372, 20130021 (2014). 24470408 10.1098/rsta.2013.0021

[b3] Bonse U. & Hart M. An x-ray interferometer. Appl. Phys. Lett. 6, 155–156 (1965).

[b4] Goetz K. *et al.* Measurements of the parameters of shell targets for laser thermonuclear fusion using an X-ray schlieren method. Kvantovaia Elektronika Moscow 6, 1037–1042 (1979).

[b5] Davis T. J., Gao D., Gureyev T. E., Stevenson A. W. & Wilkins S. W. Phase-contrast imaging of weakly absorbing materials using hard X-rays. Nature 373, 595–598 (1995).

[b6] Ingal V. N. & Beliaevskaya E. A. X-ray plane-wave topography observation of the phase contrast from a non-crystalline object. J. Phys. D Appl. Phys. 28, 2314–2317 (1995).

[b7] Wilkins S. W., Gureyev T. E., Gao D., Pogany A. & Stevenson A. W. Phase-contrast imaging using polychromatic hard x-rays. Nature 384, 335–338 (1996).

[b8] Chapman D. *et al.* Diffraction enhanced x-ray imaging. Phys. Med. Biol. 42, 2015–2025 (1997). 9394394 10.1088/0031-9155/42/11/001

[b9] Clauser J. F. inventor & Clauser J. F. assignee. Ultrahigh resolution interferometric x-ray imaging. United States patent US 5,812,629. (1998 Sep 22).

[b10] David C., Nohammer B., Solak H. H. & Ziegler E. Differential x-ray phase contrast imaging using a shearing interferometer. Appl. Phys. Lett. 81, 3287–3289 (2002).

[b11] Momose A. *et al.* Demonstration of x-ray talbot interferometry. Japanese J. Appl. Phys. 42, L866 (2003).

[b12] Pfeiffer F., Weitkamp T., Bunk O. & David C. Phase retrieval and differential phase-contrast imaging with low-brilliance X-ray sources. Nat. Phys. 2, 258–261 (2006).

[b13] Morgan K. S., Paganin D. M. & Siu K. K. W. X-ray phase imaging with a paper analyzer. Appl. Phys. Lett. 100, 124102 (2012).

[b14] Olivo A. *et al.* An innovative digital imaging set-up allowing a low-dose approach to phase contrast applications in the medical field. Med. Phys. 28, 1610–1619 (2001). 11548930 10.1118/1.1388219

[b15] Munro P. R., Ignatyev K., Speller R. D. & Olivo A. Phase and absorption retrieval using incoherent X-ray sources. Proc. Natl. Acad. Sci. USA 109, 13922–13927 (2012). 22891301 10.1073/pnas.1205396109 PMC3435200

[b16] Endrizzi M. *et al.* Hard x-ray dark-field imaging with incoherent sample illumination. Appl. Phys. Lett. 104, 024106 (2014).

[b17] Olivo A. & Speller R. A coded-aperture technique allowing x-ray phase contrast imaging with conventional sources. Appl. Phys. Lett. 91, 074106 (2007).

[b18] Endrizzi M. *et al.* Phase-contrast microscopy at high x-ray energy with a laboratory setup. Opt. Lett. 39, 3332–3335 (2014). 24876046 10.1364/OL.39.003332

[b19] Munro P. R. T., Ignatyev K., Speller R. D. & Olivo A. Source size and temporal coherence requirements of coded aperture type x-ray phase contrast imaging systems. Opt. Express 18, 19681 (2010). 20940863 10.1364/OE.18.019681 PMC3000604

[b20] Endrizzi M. *et al.* Achromatic approach to phase-based multi-modal imaging with conventional x-ray sources. Opt. Express 23, 16473–16480 (2015). 26193618 10.1364/OE.23.016473

[b21] Diemoz P. C. *et al.* X-ray phase-contrast imaging with nanoradian angular resolution. Phys. Rev. Lett. 110, 138105 (2013). 23581380 10.1103/PhysRevLett.110.138105

[b22] Diemoz P., Hagen C., Endrizzi M. & Olivo A. Sensitivity of laboratory based implementations of edge illumination x-ray phase-contrast imaging. Appl. Phys. Lett. 103, 244104 (2013).

[b23] Millard T. P. *et al.* Method for automatization of the alignment of a laboratory based x-ray phase contrast edge illumination system. Rev. Sci. Instrum. 84, 083702 (2013). 24007068 10.1063/1.4816827 PMC7116146

[b24] Endrizzi M., Basta D. & Olivo A. Laboratory-based x-ray phase-contrast imaging with misaligned optical elements. Appl. Phys. Lett. 107, 124103 (2015).

[b25] Kottler C., Pfeiffer F., Bunk O., Grünzweig C. & David C. Grating interferometer based scanning setup for hard x-ray phase contrast imaging. Rev. Sci. Instrum. 78, 043710 (2007). 17477673 10.1063/1.2723064

[b26] Koehler T. *et al.* Slit-scanning differential x-ray phase-contrast mammography: Proof-of-concept experimental studies. Med. Phys. 42, 1959–1965 (2015). 25832086 10.1118/1.4914420

[b27] Miao H. *et al.* Motionless phase stepping in x-ray phase contrast imaging with a compact source. Proc. Natl. Acad. Sci. USA 110, 19268–19272 (2013). 24218599 10.1073/pnas.1311053110 PMC3845166

[b28] Takeda M., Ina H. & Kobayashi S. Fourier-transform method of fringe-pattern analysis for computer-based topography and interferometry. J. Opt. Soc. Am. 72, 156–160 (1982).

[b29] Wen H., Bennett E. E., Hegedus M. M. & Rapacchi S. Fourier x-ray scattering radiography yields bone structural information1. Radiology 251, 910–918 (2009). 19403849 10.1148/radiol.2521081903 PMC2687535

[b30] Ge Y., Li K., Garrett J. & Chen G.-H. Grating based x-ray differential phase contrast imaging without mechanical phase stepping. Opt. Express 22, 14246–14252 (2014). 24977522 10.1364/OE.22.014246

[b31] Hagen C., Coan P., Bravin A., Olivo A. & Diemoz P. A continuous sampling scheme for edge illumination x-ray phase contrast imaging. J. Appl. Phys. 118, 054901 (2015).

[b32] Endrizzi M. & Olivo A. Absorption, refraction and scattering retrieval with an edge-illumination-based imaging setup. J. Phys. D Appl. Phys. 47, 505102 (2014).

[b33] Munro P. R. T. & Olivo A. X-ray phase-contrast imaging with polychromatic sources and the concept of effective energy. Phys. Rev. A 87, 053838 (2013).

[b34] Ignatyev K., Munro P. R. T., Speller R. D. & Olivo A. Effects of signal diffusion on x-ray phase contrast images. Rev. Sci. Instrum. 82, 073702 (2011). 21806184 10.1063/1.3606442

[b35] Endrizzi M., Oliva P., Golosio B. & Delogu P. Cmos aps detector characterization for quantitative x-ray imaging. Nucl. Instr. Meth. Phys. Res. A 703, 26–32 (2013).

[b36] Schoonjans T. *et al.* The xraylib library for x-ray–matter interactions. recent developments. Spectrochim Acta B 66, 776–784 (2011).

[b37] Wernick M. N. *et al.* Multiple-image radiography. Phys. Med. Biol. 48, 3875–3895 (2003). 14703164 10.1088/0031-9155/48/23/006

[b38] Rigon L., Arfelli F. & Menk R.-H. Generalized diffraction enhanced imaging to retrieve absorption, refraction and scattering effects. J. Phys. D Appl. Phys. 40, 3077 (2007).

[b39] Yashiro W. & Momose A. Effects of unresolvable edges in grating-based x-ray differential phase imaging. Opt. Express 23, 9233–9251 (2015). 25968757 10.1364/OE.23.009233

