# Supplementary material for: Asymmetric masks for laboratory-based X-ray phase-contrast imaging with edge illumination
Source: Sci Rep. 2016 May 5;6:25466. doi: 10.1038/srep25466 (PMC4857105; doi:10.1038/srep25466)
Supplement: Supplementary Information [file srep25466-s1.pdf]

Asymmetric masks for laboratory-based X-ray phase-contrast imaging with edge illumination

Marco Endrizzi*, **Alberto Astolfo**[1], **Fabio A. Vittoria**[1], **Thomas P. Millard**[1], **and Alessandro Olivo**[1]

[1]Department of Medical Physics and Biomedical Engineering, University College London, Gower Street, London WC1E 6BT, United Kingdom
*m.endrizzi@ucl.ac.uk

SUPPLEMENTARY INFORMATION

Illumination function shape and detector point spread function

By means of the asymmetric mask design here presented it is possible to generate illumination functions (IFs) that are relatively shifted one with respect to the other, thus enabling the acquisition of intensity projections at complementary illumination conditions without requiring movements of the imaging system. In the experimentally measured IFs (see Fig. 1(c) in the main text) a change in shape, as well as a lateral shift, can be observed. This is entirely due to the cross-talk between adjacent pixels. In the ideal case where each pixel is completely independent from its neighbours the only effect of the asymmetric design is that of shifting the IFs. When the pixels are not independent one to each other, the IFs are subject to a change in shape (possibly asymmetric). This is demonstrated by the plots in Fig. S1. Figure S1(a) shows that, by including the detector point spread function (PSF)[1] in the simulation of the experimental setup,[2] the changes in shape as well as the lateral shifts of the IFs are well matched. In addition, Fig. S1(b) shows that the ideal case simulation (no cross-talk between pixels) closely reproduces the experimental IFs acquired by using the direct conversion single-photon-counting detector.

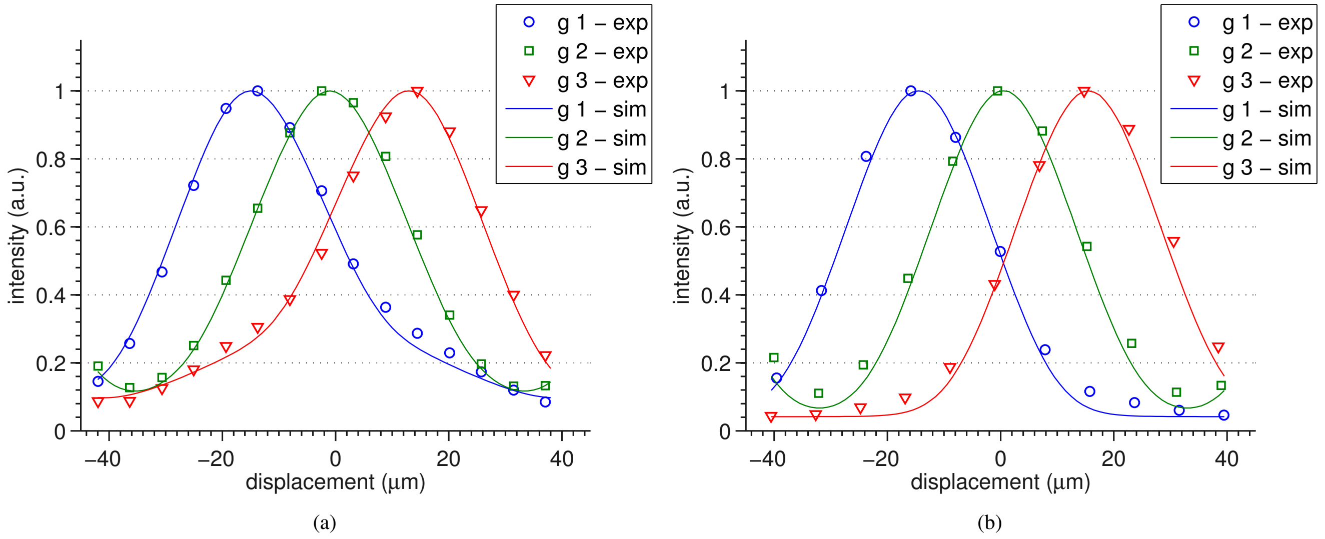

Supplementary Figure 1. Comparison of the experimental data and simulations. (a) indirect conversion detector IFs against the numerical simulation that includes the pixel cross-talk due to the shape of the detector PSF. (b) direct conversion detector IFs against the numerical simulation that assumes an ideal pixel behaviour (box PSF with no cross-talk).

It should be noted, however, that the changes in the shape of the IF are not a problem from a practical point of view. So long their shape is known, they can be measured and this information fed into the retrieval algorithm[3] leading to quantitatively accurate results.

References

1. Endrizzi, M., Oliva, P., Golosio, B. & Delogu, P. Cmos aps detector characterization for quantitative x-ray imaging. *Nucl. Instr. Meth. Phys. Res. A* **703**, 26 – 32 (2013).

2. Vittoria, F. A. *et al.* Strategies for efficient and fast wave optics simulation of coded-aperture and other x-ray phase-contrast imaging methods. *Applied Optics* **52**, 6940–6947 (2013).

3. Endrizzi, M., Basta, D. & Olivo, A. Laboratory-based x-ray phase-contrast imaging with misaligned optical elements. *Applied Physics Letters* **107**, 124103 (2015).
